# Flavin-Dependent Monooxygenases as a Detoxification Mechanism in Insects: New Insights from the Arctiids (Lepidoptera)

**DOI:** 10.1371/journal.pone.0010435

**Published:** 2010-05-03

**Authors:** Sven Sehlmeyer, Linzhu Wang, Dorothee Langel, David G. Heckel, Hoda Mohagheghi, Georg Petschenka, Dietrich Ober

**Affiliations:** 1 Biochemical Ecology and Molecular Evolution, Botanical Institute and Botanical Garden, Christian-Albrechts-Universität, Kiel, Germany; 2 Institute for Pharmaceutical Biology, TU Braunschweig, Braunschweig, Germany; 3 Department of Entomology, Max Planck Institute for Chemical Ecology, Jena, Germany; 4 Molecular Evolution, Institute of Zoology, University of Hamburg, Hamburg, Germany; CNRS UMR 8079/Université Paris-Sud, France

## Abstract

Insects experience a wide array of chemical pressures from plant allelochemicals and pesticides and have developed several effective counterstrategies to cope with such toxins. Among these, cytochrome P450 monooxygenases are crucial in plant-insect interactions. Flavin-dependent monooxygenases (FMOs) seem not to play a central role in xenobiotic detoxification in insects, in contrast to mammals. However, the previously identified senecionine *N*-oxygenase of the arctiid moth *Tyria jacobaeae* (Lepidoptera) indicates that FMOs have been recruited during the adaptation of this insect to plants that accumulate toxic pyrrolizidine alkaloids. Identification of related FMO-like sequences of various arctiids and other Lepidoptera and their combination with expressed sequence tag (EST) data and sequences emerging from the *Bombyx mori* genome project show that FMOs in Lepidoptera form a gene family with three members (FMO1 to FMO3). Phylogenetic analyses suggest that FMO3 is only distantly related to lepidopteran FMO1 and FMO2 that originated from a more recent gene duplication event. Within the FMO1 gene cluster, an additional gene duplication early in the arctiid lineage provided the basis for the evolution of the highly specific biochemical, physiological, and behavioral adaptations of these butterflies to pyrrolizidine-alkaloid-producing plants. The genes encoding pyrrolizidine-alkaloid-*N*-oxygenizing enzymes (PNOs) are transcribed in the fat body and the head of the larvae. An *N*-terminal signal peptide mediates the transport of the soluble proteins into the hemolymph where PNOs efficiently convert pro-toxic pyrrolizidine alkaloids into their non-toxic *N*-oxide derivatives. Heterologous expression of a PNO of the generalist arctiid *Grammia geneura* produced an *N*-oxygenizing enzyme that shows noticeably expanded substrate specificity compared with the related enzyme of the specialist *Tyria jacobaeae*. The data about the evolution of FMOs within lepidopteran insects and the functional characterization of a further member of this enzyme family shed light on this almost uncharacterized detoxification system in insects.

## Introduction

Flavin-dependent monooxygenases (FMOs) and cytochrome P450 monooxygenases (CYPs) are two prominent families of monooxygenases in eukaryotes [1], [2]. They catalyze the transfer of one atom of molecular oxygen to a substrate and reduce the other to water. FMO genes are found in all phyla [3]. In vertebrates, FMOs form a gene family of five similar genes. They provide an efficient detoxification system for xenobiotics, as they catalyze the conversion of heteroatom-containing chemicals from the animal's food to polar, readily excretable metabolites [4]. Yeast possesses, unlike mammals, only one FMO isoform, which has been shown to be involved in redox regulation and in the correct folding of proteins containing disulfide bonds [5], [6]. In plants, FMOs form a large gene family (29 genes in the model plant *Arabidopsis thaliana*), but information about their physiological role is sparse. For *A. thaliana*, individual FMO sequences have been related to auxin biosynthesis and pathogen defense [7]. FMOs oxygenate nucleophilic substrates that usually contain nitrogen or sulfur, such as amines, amides, thiols, and sulfides [8]. A unique feature of FMO is the catalytic cycle that forms a reactive 4α-hydroperoxyflavin intermediate as a potent monooxygenating agent before the substrate is bound to the enzyme. Like a cocked gun, this activated intermediate will readily react with all substrates that are able to access the active site [9], [10].

Our extensive knowledge about the structural and catalytic properties of vertebrate FMOs is contrasted by an almost complete lack of knowledge about this enzyme family in insects. CYP enzymes play the dominant role in drug and xenobiotic metabolism in insects [11], possibly compensating the need for FMOs in these processes. Of note, the genome of *Drosophila melanogaster* contains only two genes for FMOs [1], [12] but 90 genes for CYPs, of which 86 seem to be functional [13]. The large number of CYPs in insect genomes has been suggested to be necessary to protect the insect from the diverse array of harmful compounds in its environment [14]. This is also the case for lepidopteran species. One of the best studied examples is the CYP gene superfamily of *Papilio* butterflies (Papilionidae), which have adapted to furanocoumarins, the toxic components of their food plants [15], [16]. These toxins are degraded by inducible CYPs that are expressed in the midgut and also in the fat body [17], [18].

**Figure 1:**
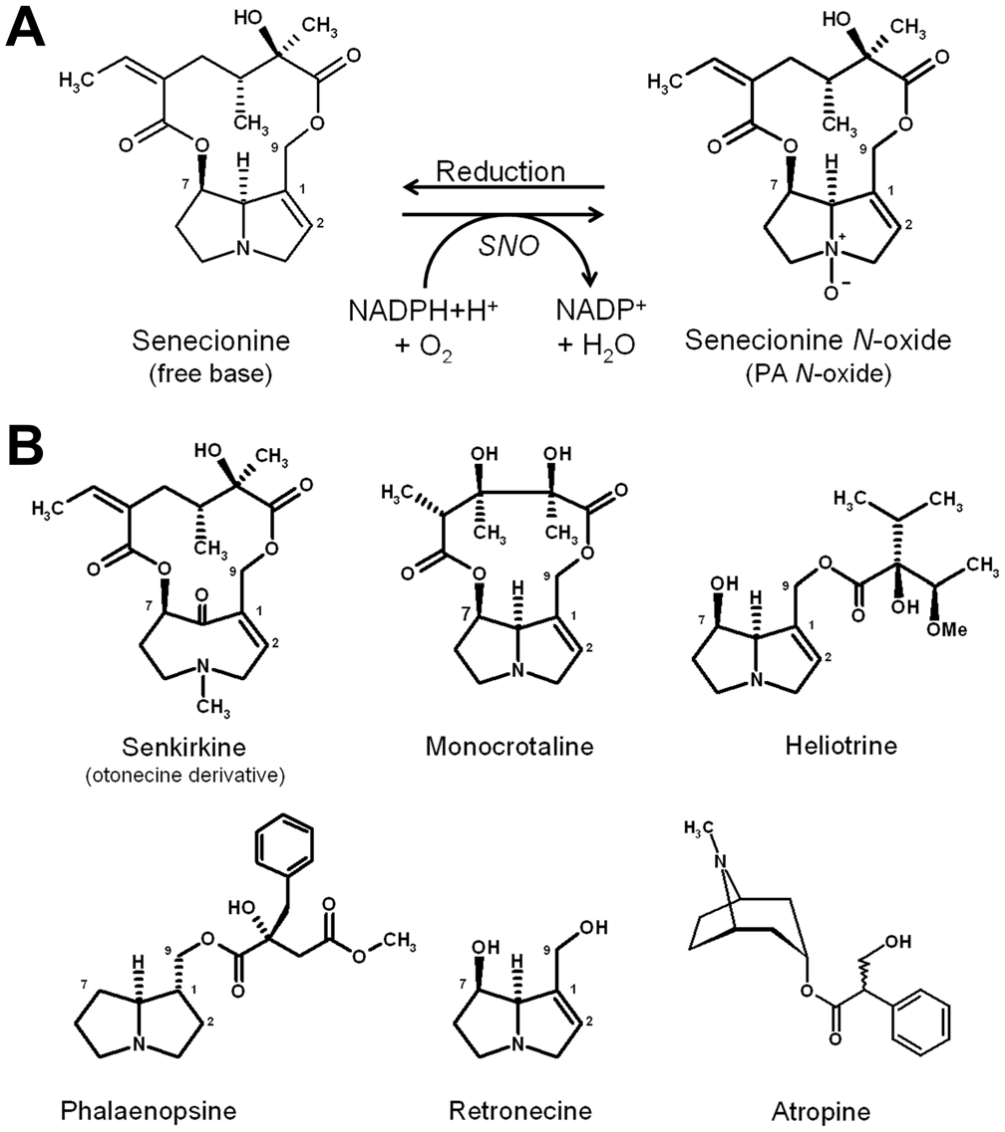
*N*-oxygenation of PAs by SNO, a flavin-dependent monooxygenase (A) and structures of selected PAs and of atropine (B). A: PAs are present in the plant mainly as *N*-oxide. After uptake in the diet, they are reduced in the gut of the herbivore to the their respective tertiary form, which is lipophilic and easily permeates membranes. In PA-adapted insects, this pro-toxic PA is efficiently converted to the respective PA *N*-oxide in the hemolymph to prevent bioactivation. B: Structures of PAs and atropine tested as substrates with recombinant SNO and PNO.

Adaptation to host plant-derived toxins has also been described for the cinnabar moth, *Tyria jacobaeae* (Arctiidae). Larvae of this species feed exclusively on tansy ragwort (*Jacobaea vulgaris*, syn. *Senecio jacobaea*), which contains toxic pyrrolizidine alkaloids (PAs) and sequester these plant toxins for their own chemical defense. PAs can exist in two transmutable forms: the pro-toxic free base (tertiary amine) and its non-toxic *N*-oxide [19], [20]. In the plant, PAs usually occur as *N*-oxides, which are easily converted to their respective free base in the reducing gut milieu of any herbivorous vertebrate or insect feeding on these plants [21], [22]. The toxicity of PAs for non-adapted insects has been shown by feeding experiments [23] and is attributable to cytochrome P450-mediated bioactivation [24]. For *Tyria*, senecionine *N*-oxygenase (SNO), a soluble enzyme located in the hemolymph, has been shown to convert the pro-toxic free base efficiently into its non-toxic *N*-oxide [22] (Figure 1). This enzyme with high substrate specificity for toxic 1,2-unsaturated PAs, is the prerequisite for sequestration of the plant-derived alkaloids by the insect. Recently, SNO has been shown to be a FMO and, until now, the only functionally characterized FMO of insect origin [1].

PA sequestration is known for many species of the tiger moth (Lepidoptera, Arctiidae), some of which use these alkaloids as a precursor for pheromone synthesis [25]. As shown for *Utetheisa ornatrix*, the PAs are acquired during the course of larval feeding and are transferred through metamorphosis to the adult stage [26]. At mating, the male advertises his PA load to the female by the PA-derived pheromone, hydroxydanaidal. Males with the highest PA load have the highest mating success and transfer a portion of their PAs via the spermatophore to the female [27], [28]. Together with the female's own load of PA, these alkaloids are passed to the eggs, protecting them against insect predators, including beetles, ants, and parasitoids [27], [29], [30].

In contrast to the specialist *T. jacobaeae*, the PA-sequestering arctiid species studied in this project are polyphagous. For *Estigmene acrea*, PAs have been shown to be important for development, as these alkaloids are used as precursors for the biosynthesis of the sex pheromone hydroxydanaidal [31], [32]. *Grammia geneura* is not known to synthesize PA-derived pheromones but benefits from sequestered PAs as defense compounds against herbivores and parasitoids [33].

Using an alignment of SNO of *T. jacobaeae* and of FMO-like sequences of the dipteran species *D. melanogaster* and *Anopheles gambiae*, we have been able to identify several new FMO sequences of arctiids and other Lepidoptera. With regard to the polyphagous species *G. geneura*, we have identified and expressed a PA-specific FMO in *E. coli* and compared its substrate specificity with the SNO of monophagous *T. jacobaeae*. Phylogenetic analysis shows that, in Lepidoptera, FMOs form three distinct sequence clusters. In one of these clusters, PA-specific FMO (PA *N*-oxygenases, PNO) originated by gene duplication early in the lineage of arctiids. These results allow a first glimpse into the hitherto untouched area of evolution and functionality of the FMO gene family in insects.

## Materials and Methods

### Insects

Larvae of *Tyria jacobaeae* were collected in The Netherlands and in the vicinity of Kiel, Germany. Larvae of *Arctia caja*, *Arctia villica*, and *Diacrisia sannio* were obtained from private breeders. Larvae of *Estigmene acrea* and *Grammia geneura* came from cultures established by Elisabeth A. Bernays and Michael S. Singer from specimens of field populations collected in southeastern Arizona, USA. All larvae were reared in the lab on a food plant mixture of *Taraxacum officinale*, *Plantago lanceolata*, and *Rubus fruticosus* or on an artificial diet [34], except for those of *T. jacobaeae*, which were exclusively reared on leaves of *Senecio jacobaea*.

### Design of degenerate primers for identification of cDNAs coding for FMO-like sequences

cDNA sequences homologous to FMOs were identified in arctiid insects by a polymerase chain reaction (PCR) approach with degenerate primers. Primers P2, P3, and P8 (Table S1) were designed based on the alignment of amino acid sequences of the SNO of *T. jacobaeae* [1] and of four putative FMOs, two each of the genome of *Drosophila melanogaster* (Acc. No. AAF47118 and AAF57364) and of *Anopheles gambiae* (Acc. No. XP_311551 and XP_311550). Later in this project, the primers P12, P17, und P29 (Table S1) were designed according to alignments that resulted from the inclusion of the amino acid sequences of the FMO of *A. caja* and *A. villica* and of the PNO of *Grammia geneura*, as identified in this project.

### Identification of cDNAs encoding FMO-like sequences of *A. caja* (AcFMO), *A. villica* (AvFMO), and *T. jacobaeae* (TjFMO)

The fat bodies of larvae of *A. caja*, *A. villica*, and *T. jacobaeae* were prepared and quickly frozen in liquid nitrogen. Total RNA was extracted by using the RNeasy Mini Kit (Qiagen) in combination with QIAshredder (Qiagen) mini spin columns. An aliquot containing 1 µg total RNA was reverse-transcribed with oligo-dT primer P1 (Table S1) by using Superscript III reverse transcriptase (Invitrogen). For identification of FMO-encoding cDNAs of *A. caja* and *A. villica*, a semi-nested PCR approach was used to amplify specific DNA fragments of ca. 900 bp in length with a constant annealing temperature of 52°C and *Taq* DNA polymerase (Invitrogen) in a total volume of 25 µl. For the first PCR, primer pair P2/P1 was used. The resulting reaction mix was diluted 1∶100 with 10 mM Tris/HCl buffer, pH 8, and used as a template for a PCR with primer pair P2/P3. The FMO-coding cDNA of *T. jacobaeae* was amplified in a nested PCR approach by using a touch-down temperature program with decreasing annealing temperature from 60°C to 45°C (0.5°C per cycle). After the first PCR with primer pair P2/P3, the reaction mix was diluted 1∶100 with 10 mM Tris/HCl buffer, pH 8, and used as template for the second PCR with primer pair P12/P29 resulting in a fragment of ca. 550 bp. For identification of the missing cDNA-ends, 3′-RACE (rapid amplification of cDNA ends) and 5′-RACE techniques were applied as described previously [35], [36] with primers P4-P7, P8-P11, and P30/P33 (Table S1) for the cDNA sequences of *A. caja*, *A. villica*, and *T. jacobaeae*, respectively. The proteins encoded by the resulting full-length sequences were denominated *Ac*FMO, *Av*FMO, and *Tj*FMO, respectively. For identification of the cDNA encoding PNO of *A. caja* (*Ac*PNO), the same strategy was used as that described for the *Ac*FMO with the following modifications: the primer pairs P2/P17 and P12/P17 were used with annealing temperatures of 54°C and 58°C for the first and second reaction of the semi-nested PCR approach, respectively. The 3′-RACE and 5′-RACE of the resulting fragment of ca. 600 bp were performed with the primers P18–P21. For identification of a cDNA encoding PNO of *G. geneura*, cDNA was prepared as described for FMO-like sequences of *A. caja* and used as template in a PCR with primer pair P12/P3 and a touch-down program with decreasing annealing temperature from 60°C to 45°C within 30 cycles. The cDNA fragment of ca. 800 bp was completed by 3′-RACE and 5′-RACE with primers P13–P16.

### Partial identification of further FMO-like cDNAs of various Arctiids

For the identification of further FMO-like cDNA sequences of *A. villica*, *Diacrisia sannio*, and *Estigmene acrea*, the same strategies were used as those described above for the identification of the full-length cDNAs of various arctiids. The PCR parameters are given in Table S2.

### Identification of FMO-like sequences of *Helicoverpa armigera* and *Bombyx mori*

FMO sequences of *D. melanogaster* and SNO of *T. jacobaeae* were used in tblastn searches of expressed sequence tag (EST) libraries of *H. armigera* and *B. mori*, and the genome sequence of *B. mori* at NCBI (http://www.ncbi.nlm.nih.gov/), KaikoBase (http://sgp.dna.affrc.go.jp/index.html), and SilkDB (http://silkworm.swu.edu.cn/silkdb/). Sequences identified from *B. mori* included FMO1 (GenBank Accession No. GU564654), FMO2 (GU564656), and FMO3 (GU564657). Genomic locations of *B.mori* FMOs were determined by using the gbrowse genome browser of Kaikobase. Coding sequences of FMO1 from clone HA-GN-M-03-libF_K16 (GU564659) and FMO3 from clone HA-PAN-384-04-libF-F3_A05 (GU564662) were obtained from cDNA libraries constructed from *H. armigera* larvae by Heiko Vogel, Max Planck Institute for Chemical Ecology. The sequence of FMO2 from clone 28d18 (GU564660) was obtained from a cDNA library constructed from *H. armigera* larval midgut by Vladimir D. Grubor, University of Melbourne [37].

### Sequence analysis

cDNA sequences were analyzed by using the ProtParam tool [38] for prediction of various protein properties, by using the signalP3.0 server [39] for prediction of signal peptides and their cleavage sites, by using the PSORT II and the TargetP1.1 server [40], [41] for the detection of sorting signals and subcellular localizations, and by using the TMHMM2.0 Server [42] for the prediction of transmembrane helices. To generate an alignment of amino acid sequences, ClustalX [43] was used before phylogenies were estimated with the following software of the PHYLIP program package [44]: PROTDIST with the Jones-Taylor-Thornton matrix in combination with NEIGHBOR for neighbor-joining analyses [45] and SEQBOOT and CONSENSE to estimate bootstrap values. Accession numbers of sequences taken from the database are given in Table S3.

### Heterologous expression of cDNA encoding PNO of *Grammia geneura*

The open reading frame (ORF) of the PNO of *Grammia geneura* was analyzed for the presence of an *N*-terminal signal peptide by using the SignalP3.0 server [39]. For amplification of the whole ORF without the signal peptide, a pair of gene-specific primers was constructed that contained restriction sites for subcloning in addition to an artificial initiation codon (P34, *Nde*I) and the stop codon behind an artificial sequence encoding six histidine residues (P35, *Bam*HI). The fragment resulting from amplification with AccuTaq LA DNA Polymerase (Sigma) of the oligo(dT)_17_ primed cDNA of *G. geneura* as the template was *Nde*I/*Bam*HI-digested and ligated into a *Nde*I/*Bam*HI-linearized pET3a vector (Novagen) for expression with the T_7_ polymerase system [46]. Positive clones were identified as described previously for SNO of *T. jacobaeae* [1]. Expression in *E. coli* BL21(DE3) (Stratagene) harboring the pRDKJG plasmid [47], was achieved in a modified lysogeny broth (LB) medium pH 7.5 containing 2.5 mM betaine hydrochloride and 0.8 M sorbitol at 4°C for 96 h.

### Semiquantitative reverse transcription PCR

Per sample, 1 µg total RNA was used as a template for cDNA synthesis with an oligo-dT primer (P1) by using Superscript III RT (Invitrogen, Carlsbad, CA). PCR was performed with *Taq* DNA polymerase (Invitrogen) and the following temperature program: 4 min at 95°C initial denaturation, 46 cycles with an annealing temperature of 65°C, and elongation at 72°C for 2 min. The primer pairs specific for *Tj*SNO (P36/P37) and *Tj*FMO (P38/P39) resulted in fragments of ca. 1400 bp. Aliquots were taken at intervals of 3 cycles starting after cycle 28.

### Enzyme assay

Enzyme activity of PNOs was assayed photometrically or by qualitative tracer assays as described previously [1]. Briefly, for the photometric assay, reactions were set up in a total volume of 300 µl 10 mM potassium phosphate pH 8 and 120 µM NADPH. After preincubation for 3 min at 37°C, the reaction was started by addition of 170 µM PA substrate and incubated at 37°C. The reaction was followed by the decrease of NADPH absorption at 340 nm with an Ultrospec 2100 pro UV/Visible Spectrophotometer (GE Healthcare). For the tracer assays, the volume of the reaction mixture was reduced to 50 µl. The amount of radioactively labeled PA *N*-oxide formed from tertiary PA was analyzed by thin-layer chromatography (TLC). Aliquots of 12.5 µl were therefore taken from the reaction mixture at time intervals between 3 to 30 min to ensure linearity of product formation and applied to silica gel 60 F_254_ TLC plates (Merck, Darmstadt). Enzyme activity was calculated from the substrate (PA)/product (PA *N*-oxide) ratio after separation by using the solvent system dichloromethane∶methanol∶ammonium hydroxide (25%) at a ratio of 80∶20∶3. Radioscans were performed by means of a radioactivity thin-layer analyzer (RITA, Raytest, Straubenhardt, Germany). PAs applied as enzyme substrates were obtained as described [22].

## Results

### Identification of cDNA sequences encoding FMO-like proteins from Arctiids

**Figure 2:**
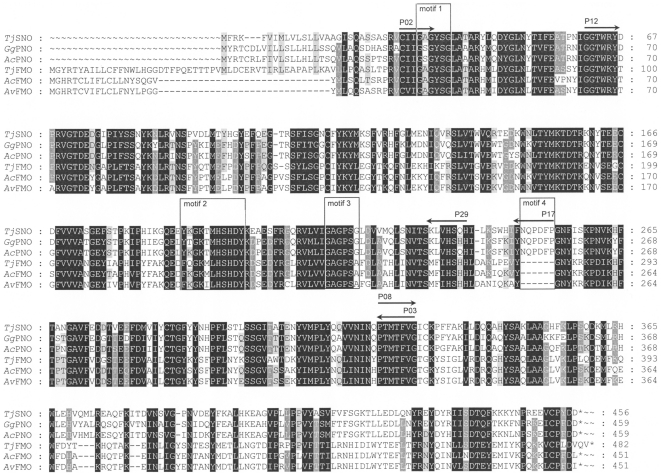
Alignment of the amino acid sequences of FMOs of various arctiid species. Motif 1, FAD-binding site; motif 2, FMO-identifying sequence; motif 3, NADPH-binding site; motif 4, insertion of six amino acids characteristic for sequences belonging to the putative PNO cluster; *Tj*SNO, *T. jacobaeae* SNO; *Gg*PNO, *G. geneura* PNO; *Ac*PNO, *A. caja* PNO; *Tj*FMO, *T. jacobaeae* FMO; *Ac*FMO, *A. caja* FMO; *Av*FMO, *A. villica* FMO.

For identification of cDNA sequences encoding FMOs from Arctiids, species were selected that were closely related to the specialist *T. jacobaeae*. These species included the polyphagous species *Arctia caja*, *Diacrisia sannio*, *Estigmene acrea*, and *Grammia geneura*, for all of which an association with PA-containing plants was previously described [48]. For the species *Arctia villica*, the PA association was confirmed by the ability to *N*-oxygenize tertiary PAs. Senecionine *N-*oxide was detected after incubation of the hemolymph with [^14^C]senecionine as the sole substrate in an assay for PNO activity (data not shown). For cDNA identification by a reverse transcription (RT)-PCR approach, an alignment of FMO-like sequences from insects was used to design degenerate primers. Two sequences identified within each of the genomes of *Anopheles gambiae* and *Drosophila melanogaster*, respectively, and the SNO of *T. jacobaeae* (*Tj*SNO) were the only FMO-like sequences of insect origin that were available in this phase of our project. The degree of identity between these sequences at the amino acid level was between 33% and 56%. Conserved sequence motifs that were appropriate for primer design encompassed the FAD- and NADPH-binding sites (motifs 1 and 3 in Figure 2) and an additional conserved sequence stretch in the C-terminal part of the sequence (amino acid 323–329 of *Tj*SNO). Later in this project, after identification of three FMO-like sequences from *A. caja*, *A. villica*, and *G. geneura*, we were able to improve the quality of the alignment and of the degenerate primers. Of all species, except for *G. geneura*, we identified two distinct cDNA sequences of which the derived amino acid sequences showed a high degree of identity to other FMO sequences in the database. The complete ORF was determined for the cDNAs encoding the PNO of *G. geneura* (*Gg*PNO), a putative PNO of *Arctia caja* (*Ac*PNO), and a FMO-like sequence of unknown function of each, *T. jacobaeae* (*Tj*FMO) *A. caja* (*Ac*FMO), and *A. villica* (*Av*FMO) (Table 1). In addition, partial sequences were isolated from *Diacrisia sannio* (*Ds*FMO and *Ds*PNO) and *Estigmene acrea* (*Ea*FMO and *Ea*PNO) and an additional sequence *A. villica* (*Av*PNO). Of those sequences for which we were able to identify the complete coding region, the properties of the cDNA and the encoded proteins are summarized in Table 1. Figure 2 shows that the ORFs of these sequences possess all three characteristic motifs of FMO sequences, i.e., the nucleotide-binding domains that stabilize the binding of FAD (consensus: GxGxxG) and NADPH (consensus: GxGxx(A/G)), respectively, and the FMO-identifying sequence (consensus: FxGxxxHxxx(F/Y)). Computer-based sequence analyses predicted *N*-terminal signal peptides for the vesicular pathway and a lack of a C-terminal membrane anchor, properties previously identified for the senecionine *N*-oxygenase of *T. jacobaeae* [1].

**Table 1:** Characteristics of FMO-like sequences identified from three Arctiid species.

		length of cDNA [bp]	ORF [bp]	5′-UTR [bp]	3′-UTR [bp]	length of SP [bp]	MW + SP	[kDa] − SP	IEP − SP	membrane anchor	localization
*Tj*SNO^1^	*T. jacobaeae*	1701	1371	81	249	22	52.2	49.8	6.4	no	extracellular
*Gg*PNO	*G. geneura*	1775	1380	80	315	23	52.7	50.1	6.4	no	extracellular
*Ac*PNO	*A. caja*	1938	1380	64	494	23	52.4	49.8	6.5	no	extracellular
*Tj*FMO	*T. jacobaeae*	1719	1452	84	186	18 (21)	54.9	52.7	6.4	no	extracellular
*Ac*FMO	*A. caja*	1664	1356	83	225	18	51.4	49.4	7.0	no	extracellular
*Av*FMO	*A. villica*	1675	1356	90	229	18	51.4	49.3	6.7	no	extracellular

ORF, open reading frame; UTR, untranslated region, (+/−) SP, (with/without) *N*-terminal signal peptide; MW, molecular weight; IEP, isoelectric point.

1 data taken from [1].

### Heterologous expression of recombinant proteins

The heterologous expression of the SNO of *T. jacobaeae* resulted in the formation of an insoluble and inactive protein [1]. Despite the finding that protein solubilization and subsequent renaturation resulted in an active protein, the specific activity remained low. Therefore, we tried to improve the expression system by using Sf9 insect cells, a system based on pupal ovarian cells of *Spodoptera frugiperda*, a noctuid lepidopteran species related to the Arctiids. Using this system, we were able to detect active SNO in the medium supporting the functionality of the predicted *N*-terminal signal peptide, but the yield of protein was too low for further biochemical characterization. Insufficient protein yield was also the problem when using a yeast expression system (data not shown). Finally, by modifying a method described by Blackwell and Horgan [49], we succeeded in expressing PNO of *G. geneura* without the signal peptide in at least a partially soluble and active form. Therefore, we added betaine to the medium and promoted its uptake by osmotic stress by sorbitol. Further improvements were achieved by coexpression with the *E. coli* chaperones DnaK/DnaJ/GrpE by using the plasmid pRDKJG [47] and by reducing the expression temperature to 4°C. The purified PNO of *G. geneura* showed a specific activity of 55 nkat/mg with senecionine as substrate.

### Substrate specificity of recombinant PNO of *G. geneura*

The substrate specificity of the PNO of the generalist *G. geneura* was characterized to compare it with the data described previously for SNO of the specialist *T. jacobaeae* [1], [22]. Therefore, PAs of the various structural types were tested in addition to some other alkaloids and to substrates of mammalian and yeast FMO (Table 2). The data show that all tested PAs, with exception of the otonecine derivative of senecionine, viz., senkirkine, were substrates for the PNO of *G. geneura*. The most obvious differences from the SNO of *T. jacobaeae* was the ability of the PNO to *N*-oxygenize phalaenopsine, a 1,2-saturated PA, and atropine. Dimethylaniline, as a typical substrate for mammalian FMO, necine bases, and nicotine was neither accepted by the specialist's enzyme of *T. jacobaeae*, nor by the generalist's enzyme of *G. geneura*. Of note, the enzyme of *G. geneura* showed a low but unequivocal activity with glutathione as substrate.

**Table 2:** Substrate specificity of native*Tj*SNO and recombinant *Gg*PNO.

	activity [%]	
Substrate	SNO of *T. jacobaeae* 1	PNO of *G. geneura*
**Pyrrolizidine alkaloids**		
Senecionine type		
Senecionine	100	61
Seneciphylline	95	59
Senkirkine	n.d.	n.d.
Monocrotaline type		
Monocrotaline	92	100
Axillarine	74	69
Lycopsamine type		
Heliotrine	25	49
Rinderine	23	43
Phalaenopsine type		
Phalaenopsine	n.d.	19
**Other substrates**		
Retronecine	n.d.	n.d.
Supinidine	n.d.	n.d.
Atropine	n.d.	21
Nicotine	–	n.d.
Dimethylaniline	n.d.	n.d.
L-Cysteine	n.d.	n.d.
Cysteamine	n.d.	n.d.
Glutathione	n.d.	41

Relative acitivities refer to 100% values of 77.6 nkat/mg with senecionine for *Tj*SNO and 90.2 nkat/mg with monocrotaline for *Gg*PNO. n.d., not detectable, –, not tested.

1 according to [1], [22].

### Tissue-specific expression of FMOs in insects

**Figure 3:**
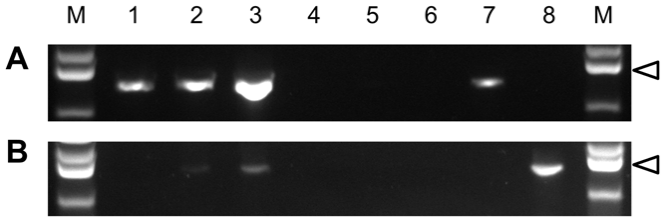
Tissue-specific expression of SNO (A) and FMO (B) of *T. jacobaeae*. Semiquantitative reverse transcription PCR was performed with total RNA of various tissues of *T. jacobaeae* larvae. Aliquots were taken after 34 and 43 cycles of amplification with primers specific for *Tj*SNO (A) and *Tj*FMO (B), respectively. M, 100-bp DNA ladder (Fermentas) with the 1500-bp fragment labeled by an arrowhead; lane 1, head; lane 2, integument; lane 3, fat body; lane 4, hemolymph; lane 5, gut; lane 6, negative control (H_2_O); lane 7 and lane 8, positive controls (0.5 pg of plasmid carrying the full-length cDNA encoding *Tj*SNO and *Tj*FMO, respectively).

The SNO of *T. jacobaeae* is a soluble protein present in the hemolymph [22]. As a signal peptide was identified at the *N*-terminus of the respective cDNA [1], a semiquantitative RT-PCR approach was used to identify the tissue expressing the transcript of PA-specific SNO in comparison with the FMO of unknown function. The larvae were dissected, and the various tissues used separately for total RNA extraction. As shown in Figure 3, the *N*-oxygenase transcript was detectable in the fat body, the tissue that synthesizes and secretes proteins of the hemolymph [50]. In addition, a signal was detectable in the integument that might have been attributable to contamination of the sample tissue by adhering fat body tissue, and a signal was detected in the head of the larvae. Cloning and sequencing of the PCR products of the head and the fat body sample revealed that both transcripts were identical at the nucleic acid level.

### PA-specific N-oxygenases form a separate cluster within lepidopteran FMO1

**Figure 4:**
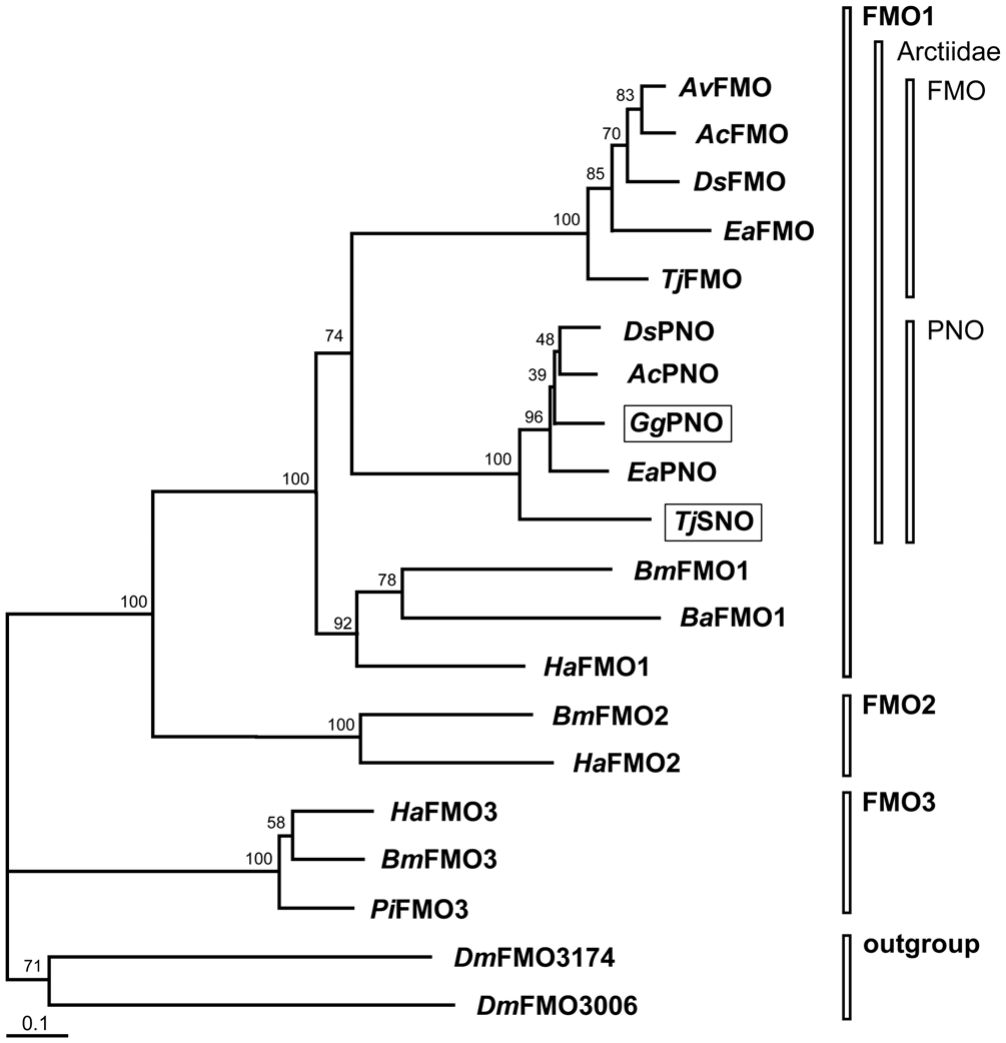
Rooted neighbor-joining tree of amino acid sequences derived from cDNA encoding lepidopteran FMOs with two sequences of *D. melanogaster* as the outgroup. The framed sequences were experimentally characterized as SNO and PNO, respectively, whereas the others should be regarded as putative FMO-coding cDNA. Branch lengths are proportional to the number of substitutions per site (scale: 0.1 substitutions per site). Bootstrap proportions resulted from 1000 replicates. *Ac*, *Arctia caja*; *Av*, *Arctia villica*; *Ba*, *Bicyclus anynana*; *Bm*, *Bombyx mori*; *Dm*, *Drosophila melanogaster*; *Ds*, *Diacrisia sannio*; *Ea*, *Estigmene acrea*; *Gg*, *Grammia geneura*; *Ha*, *Helicoverpa armigera*; *Pi*, *Plodia interpunctella*; *Tj*, *Tyria jacobaeae*.

For phylogenetic analysis, we used the arctiid sequences identified in this project in combination with selected FMO-like sequences from Lepidoptera available in the databases. EST sequences of *Bicyclus anynana* (Nymphalidae) and *Plodia interpunctella* (Pyralidae) were assembled to deduce the encoded amino acid sequences. Three FMO-like sequences were taken from the *Bombyx mori* (Bombycidae) genome database (SilkDB, http://silkworm.genomics.org.cn/). Additionally, we included three sequences from *Helicoverpa armigera* (Noctuidae) that were identified from a cDNA library of larval midgut [37] and of entire larvae (H. Vogel, unpublished results), respectively. To avoid misleading results attributable to missing N-terminal and C-terminal ends of sequences, only the central part of the alignment was used in which gaps of unidentified sequences had to be filled with replacement characters within the sequences of *P. interpunctella*, *B. anynana*, and the FMO of *E. acrea* (*Ea*FMO). The alignment is available as Figure S1. A neighbor-joining tree with two FMO-like sequences from the genome of *Drosophila melanogaster* as outgroup is shown in Figure 4. The branching pattern shows that lepidopteran FMO-like sequences occur in three well-supported clusters. The presence of one of the three FMO-like sequences of *Bombyx mori* in each of these clusters suggested that FMO in Lepidoptera formed a gene family of three members that we termed FMO1, FMO2, and FMO3 in analogy to the mammalian FMO gene family consisting of five members (FMO1 to FMO5) [51]. FMO3 sequences are only distantly related to lepidopteran FMO1 and FMO2. The closer relationship between FMO1 and FMO2 suggested by the tree is supported by the observation that both genes sit next to each other (tail-to-tail) on chromosome 25 of the *Bombyx* genome, indicating that they originated by a gene duplication event. All arctiid FMOs were identified within this project group with FMO1 and split into two distinct and well-supported clusters of paralogous sequences. In both clusters, the sequences identified from *T. jacobaeae*, i.e., *Tj*SNO and *Tj*FMO, respectively, were each sisters to all other sequences of the respective cluster. This branching pattern is supported by the classification of *T. jacobaeae* into the tribe Callimorphini (subfamily Arctiinae), in contrast to the other arctiid species of this study, which belong to the tribe Arctiini [52]. One of the two arctiid FMO clusters contained the functionally characterized SNO of *T. jacobaeae* [1] and the PNO of *G. geneura*.

## Discussion

Insects experience a wide array of chemical pressures from plant allelochemicals and pesticides and have developed several effective counterstrategies to cope with these toxins [53]. Among these, CYPs appear to have a key role in plant-insect interactions [11]. In mammals, the P450 system of xenobiotic detoxification is supplemented by the FMO gene family, which consists of five genes [54], [55]. Only recently, we have been able to identify and to characterize functionally the first FMO of insects. The cinnabar moth *Tyria jacobaeae* (Lepidoptera, Arctiidae), an insect specialized for plants containing toxic PAs, has evolved an FMO for the modification and storage of these plant-derived toxins [1]. Here, we describe the identification of several members of the FMO-gene family in lepidopteran species with a focus on sequences of the tiger moth family (Arctiidae), which recruited FMO-encoding genes for adaptation to PA-containing plants by means of a gene duplication early in its lineage. The invention of this new class of FMO was the prerequisite for these insects (i) to feed, unrivaled, on PA-containing plants, (ii) to convert these plant toxins to pro-toxins and to sequester them for their own chemical defense, and (iii) to use them in certain cases for the biosynthesis of sex pheromones.

### Optimization of heterologous expression of soluble and active PNO in *E. coli*

Using an alignment of FMO sequences of *D. melanogaster*, *A. gambiae*, and *T. jacobaeae* for degenerate primer design, we have been able to identify five full-length cDNA sequences of arctiid FMOs (Table 1) in addition to several sequence fragments. All sequences have been classified as FMO, because of their sequence similarities and characteristic sequence motifs, i.e., two dinucleotide-binding signatures (Rossman folds) for FAD and NADP and the FMO-identifying motif FxGxxxHxxx(Y/F) [2], [56]. In addition to these three fingerprint sequences for FMO (motifs 1 to 3 in Figure 2), we have identified another motif (motif 4 in Figure 2) representing an insertion of six amino acids that is most characteristic for these sequences in the PNO cluster of Arctiid sequences (Figure 4). A primer constructed on this sequence motif (primer P17, Figure 2) has enabled us to restrict the amplification of FMO homologs to sequences belonging to this sequence cluster. We have used this strategy successfully for the identification of sequence fragments of the putative PNO of *A. caja*, *A. villica*, *D. sannio*, and *E. acrea*. The PNO identified from the generalist *G. geneura* is also characterized by this sequence insertion and has been selected for heterologous expression and functional characterization. In a previous study, attempts to express the SNO of *T. jacobaeae* heterologously in *E. coli* were hampered by the formation of inclusion bodies. For biochemical analysis, the inclusion bodies had to be solubilized under denaturing conditions followed by a renaturation procedure requiring several dialysis steps [1]. Nevertheless, the yield of active enzyme was low in comparison with activities observed for the native enzyme (0.5 nkat/mg in comparison to 77.6 nkat/mg [1]). As expression of *Gg*PNO in *E. coli* also results in inclusion body formation, we have optimized the expression system. The supplementation of the medium with betaine in the presence of sorbitol, the coexpression of *E. coli* chaperons that have proved to be helpful previously [57], and a drastic reduction of the expression temperature to 4°C have all led to the expression of soluble and active protein. The specific activity of about 55 nkat/mg almost equals the value of 77.6 nkat/mg that has been described for the native SNO of *Tyria jacobaeae* [1].

### *Gg*PNO encodes a PA *N*-oxygenase with extended substrate specificity

The SNO of the specialist *T. jacobaeae* (*Tj*SNO) is characterized by a high substrate specificity for PAs that are toxic because of the following structural features: (i) an 1,2-double bond in the ring system, (ii) an allylic esterified hydroxyl group at C9, and (iii) a free or esterified hydroxyl group at C9 [22]. The same high specificity has been established for the recombinant SNO that is heterologously expressed in *E. coli* [1]. In contrast, the PNO of *G. geneura* accepts a wider range of substrates. In addition to the alkaloids accepted by *Tj*SNO, the 1,2-saturated PA phalaenopsine is *N*-oxygenized. The only PA that is not accepted by *Grammia* PNO and *Tyria* SNO is senkirkine, an otonecine derivative that cannot be *N*-oxygenized because of a methyl group at the ring-bound nitrogen. Feeding experiments of *G. geneura* have shown that senkirkine cannot be detoxified by *N*-oxidation and is neither sequestered nor metabolized [58]. 1,2-saturated PAs are devoid of the characteristic double bond, and therefore, they are regarded as non-toxic at least as far as bioactivation-mediated toxicity is concerned. However, the observations that 1,2-saturated PAs are accumulated in the plant preferentially in reproductive and young tissues [59], [60], [61] and that these structures might mediate antifeedant activity and neurotoxic effects [62], [63] suggest an ecological role for these alkaloids. The finding that phalaenopsine is *N-*oxygenized by *Grammia* PNO is in good agreement with the described ability of the larvae of *G. geneura* to sequester and metabolize these 1,2-saturated PAs into insect PAs. The observation that these insect PAs are transferred via metamorphosis to the adult stage has been interpreted as support for their ecological role for the insect, most probably in the chemical defense of the insect [58]. Of note, atropine, a tropane alkaloid produced by certain solanacous plants as part of their chemical defense against herbivores [64] is also accepted by *Grammia* PNO as a substrate. This wide substrate specificity is in accord with the finding that *Grammia* feeds as a generalist on a wide variety of food plants. Approximately 80 different species of about 50 taxonomically disparate families have been counted by Singer et al. [65], of which several are avoided by other generalist insects because of their toxicity, including species containing various types of PAs. Differences with respect to substrate specificity have also been described for CYPs as counterdefense enzymes of the specialist *Papilio polyxenes*, which feeds exclusively on furanocoumarin-containing plants and of the generalist *Helicoverpa zea*, which feeds on hundreds of types of host plant [66]. The toxicological challenge of generalized feeding is considerable with respect to the tremendous diversity of plant defense compounds. Specialization on a narrow range of host plants is interpreted as an adaptative strategy to plant toxins, involving more specialized detoxification enzymes [66]. Future research has to show the number and kind of enzymes that are involved, in addition to PNO, in the detoxification of plant-derived toxins in polyphagous Arctiids.

Remarkably, *Grammia* PNO also accepts glutathione, a substrate described for yeast FMO but that is not accepted by the SNO of *Tyria*. By oxidation of glutathione and other biological alcohols, yeast FMO provides the oxidizing equivalents that are essential for the proper folding of disulfide-containing proteins at the endoplasmic reticulum [67], [68]. Currently, we do not know whether this activity is a unique feature of the PNO of *Grammia*, or whether this conversion is an inherent activity of lepidopteran FMOs, suggesting a similar physiological role for lepidopteran FMOs as described for yeast and postulated for the FMOs of mammals and of *Trypanosoma cruzi* [69], [70]. The observation that the SNO of *T. jacobaeae* is expressed not only in the fat body, but also in the head of the larvae, suggests that the respective genes are pleiotropic and not only involved in PA *N*-oxygenation.

### Duplication of a FMO-encoding gene as a key innovation in the Arctiids for adaptation to PA-containing plants

The identification of several lepidopteran FMO-like sequences in this project has enabled us to construct a neighbor-joining tree of this gene family present in this order of insects. The branching pattern of this tree shows three well-supported clusters that we have named FMO1 to FMO3, each containing one of the three FMO-coding sequences present in the *Bombyx mori* genome. Incorporation of EST sequence data of other lepidopteran species available in the databases supports these three clusters (data not shown). Two of these EST sequences that have shown the lowest degree of missing sequence in our alignment have been included in the phylogenetic analysis. The finding that the genomes of *D. melanogaster* and *A. gambiae* contain only two FMO-like sequences that do not cluster with FMO sequences of the Lepidoptera suggests that lepidopteran FMO form a lineage-specific group. A similar observation is described by Hao et al. [3] who has reconstructed a phylogeny of 104 FMO sequences of 34 species belonging to various metazoan phyla. The mammalian FMOs encompassing the five types FMO1–FMO5 show a monophyletic origin, well separated from the clades containing the fish FMO or the invertebrate FMO-like sequences. Therefore, the different lineages of animals do not have truly orthologous genes. Instead, diversification of the *fmo* genes occurred independently in the lineages by gene duplications, resulting in gene copies some of which were lost again, with others evolving different functions and metabolizing different substrates [3], [71]. Within the Lepidoptera, the gene duplication that resulted in the origin of FMO1 and FMO2 is well supported by the position of both genes close to each other on the chromosome 25 in the *B. mori* genome. Another gene duplication seems to be specific for the lineage of the Arctiids and has resulted in a cluster of FMO sequences of unknown function and in a separate cluster, encompassing sequences of which two have been shown to be involved in the *N*-oxygenation of plant-derived PAs, i.e., the SNO of *T. jacobaeae* and the PNO of *G. geneura*. Therefore, this gene duplication can be interpreted as a “key innovation” within this lineage according to the interpretation of Berenbaum et al. [72], it being the prerequisite for the evolution of the biochemical basis for the multifaceted adaptations of tiger moths to PA-containing plants. Indeed, in 1999, Weller et al. [52] postulated that the ability to sequester PAs from the larval diet should have arisen at an ancestral node early in the Arctiid family.

Of note, all FMO-like sequences that have been identified from arctiid species can be grouped into the two arctiid-specific clusters within the FMO1 group. No single sequence has been identified that clusters with FMO2 and FMO3 of other lepidopteran species, although the degenerate primers used for our approach were at least at the beginning of our study, not specific for lepidopteran FMO. For the design of the degenerate primers, we have used an alignment of only one lepidopteran FMO (*Tj*SNO) and of four FMOs from two dipteran genomes. Work is in progress to test whether this arises from using mainly fat body tissues for cDNA preparations or whether the arctiids are indeed devoid of any sequences orthologous to FMO2 and FMO3 of *B. mori* because of a loss of the respective genes. In this regard, the branch length of the arctiid FMO sequences are notably longer than those of the PNO-sequence cluster or of other lepidopteran FMO1, suggesting a higher substitution rate within these FMO-coding sequences. A challenge for the future will be to assign a specific function to this arctiid-specific sequence cluster and to compare it with the FMO1 sequences of other lepidopteran species.

## Supporting Information

Figure S1 Amino acid alignment of flavin-dependent monooxygenases of various lepidopteren species.
(0.04 MB PDF)

Table S1 Sequences of primers used for the identification and cloning of cDNAs of flavin-dependent monooxygenases of the Lepidoptera.
(0.02 MB PDF)

Table S2 PCR-based strategy for identification of partial FMO-like sequences of various lepidopteren species.
(0.01 MB PDF)

Table S3 Accession numbers of all nucleotide sequences that have been identified within this project and that have been taken from the databases.
(0.01 MB PDF)
