# Supplementary material for: Flavin-Dependent Monooxygenases as a Detoxification Mechanism in Insects: New Insights from the Arctiids (Lepidoptera)
Source: PLoS One. 2010 May 3;5(5):e10435. doi: 10.1371/journal.pone.0010435 (PMC2862711; doi:10.1371/journal.pone.0010435)
Supplement: Figure S1 — Amino acid alignment of flavin-dependent monooxygenases of various lepidopteren species. (0.04 MB PDF) [file pone.0010435.s001.pdf]

```
                                       10        20        30        40        50        60        70        80        90       100
                              ....|....|....|....|....|....|....|....|....|....|....|....|....|....|....|....|....|....|....|....|
BmFMO2LR                      GGTWRYDPRVGTDENGLPLHTSMYKHLHTNLPKPTMELRGFPLPDGI-PSFPSWKIYYDYLKDYAKHFDIEKYIQFRHNVTLV----RREQNVWKVTHEH
HaFMO2LR                      GGTWRYDTHVGTDENGQPLHTSMYKYLRTNLPKAPMEMRGFPLPDYL-PSYPTGRDFYHYLEECVDRLDIKKYIKFLHAVVSV----RRINEVWKVKYEH
BmFMO1LR                      GGTWHFDPHVGTDEDGLPVFSSMYNDLRTNTPRQTMEYYDFPFPEG-TPSYPSATCFLDYLKSFVKHFDLLSHIQLRSLVTSV----KWAGNHWNLTYTK
HaFMO1LR                      GGTWRFDPHVGTDEDGLPLFTSMYKNLRTNTPRQTMEYAGFPFPEG-TPSYPTGPCFYKYLQHFAKHFELMNNIQLQSYVNLV----KWAKDHWEVTYTK
Bicyclus-FMO1-PepPart         XXXXXXXXXXXXXXXXXXXXXXXXXXXXXXXXXXXXXXXXXXXXXXXXXSYPSGPCFFKYLKSFVNKFDLLPNIQVRSLVTSV----TWADDHWNLTYFR
AcFMO1pGEMproof+S             GGTWRYTPRVGTDEYGAPLFTSAYKDLRTNSFYPTMELPDYPFPAGPVSSFLSGPCIYKYLEGYTKQFNLEKYIQFRSLVTNV----EKVGDNWKVTYMK
AvFMO1hypLR                   GGTWRYTARVGTDEYGAPLFTSAYKDLRTNSFYPTMELPDYPFPAGPVSSFLSGPCIYKYLEGYTKQFNLEKYIQFRSLVTSV----EKVGDNWNVTYMK
dsannioFMOint_3raceLR         GGTWSYTPRVGTDEYGAPLFTSSYKDLRTNSFYQSMELPDYPFPAGPVSSFLSGPCIYKYLEGFTKQFNLEKYIRFRSLVNSV----EKVGDNWNVTYMK
TjFMO2pGEMproof+S             GGTWKYTPRVGTDENGAPLFTSAYKNLRTNSFYQTMEFPDYPFPQGS-SSYLSGPCIYKYLQGYTKQFNLEKHIKFQSLVTSV----ERVGDMWNVTYMK
eacreaFMOint_3race_tempLR     XXXXXXXXXXXXXXXXXXXXXXXXXXXXXXXXXXXXEFPDYPFPTGVLSSFLSGPCIYKYLEGYTEQFNIAKYIQFRSLVTNV----EKVDDNWKVTYMK
AcSNOoSproofLR                GGTWRYDPRVGTDEDGLPIFSSQYKNLRINSPYKIMEFHDYSFPEG-TRSFISGGCFYKYMKSFVRHFGLMDNIQVQSLITWV----EWTGYSWNLTYMK
dsannioSNOint_3raceLR         -GTWRYDPRVGTDEDGIPIFSSQYKNLRTNSPYKIMEFHNYSFPED-TRSFISGGCFYKYMKSFVRHFGLMDNIQVQSLVTWV----EWTGDSWNLTYMK
GgSNOoSproofLR                GGTWRYDPRVGTDEDGLPIFSSQYKYLRTNSPYKIMEFHDYPFPEG-TRSFITGGCFYKYMKSFVRHFKLMDNIQVQSLVTWV----EWTGDNWKVTYMK
eacreaSNOint+3raceLR          GGTWRYDPRVGTDEDGLPIFSSQYKNLRTNSPFKIMEFHNYSFPEG-TRSFVTGGCFYKYMKSFVRYFGLMENIQVQSLVTWV----EWTGDSWNLTYMK
NoxTyriaproofLR-S             GGTWRYDPRVGTDEDGIPIYSSNYKNLRVNSPVDLMTYHGYEFQEG-TRSFISGNCFYKYMKSFVRHFGLMENIQVRSLVTWV----QRTEDKWNLTYMK
BmFMO3LR                      GGTWVYTEKVGYDDFGLPIHSSMYKSLRTNLPKEIMGFPDFPVPES-EKSYLPAKEMLSFLQLYADKHQVTDRINFNHHVNLVIPKAGPSGELWDVSFKN
HaFMO3bLR                     GGTWVYTENVGYDDFGLPIHTSMYKSLRTNLPKEIMGFPDFPVPES-EQSYLPAKDMLAFLKLYADKHGVTEKIKFSHHVQLVIPKQGPSGELWDVSYKN
Plodia-FMO3-PepPart           GGTWVYTEKVGYDDFGLPIHTSMYKSLRTNLPKEVMGFPDFPVPES-EKSYLPAKEMLAFLQLYADKHGVTSHIKYNQHVQLVIPKAGPAGELWDVSFKD
FMO3006                       GGTWVYNEATGAVN-GIDVHSSMYKNLRTNLPKEVMGFPDFEIGAN-EASYVRSDEICDFLNQYANHFDLKKHIKFDSYVIRVLQRK----TKWQVLFKD
FMO3174                       GGTWIFSEEMPKDE-YDEVHSSMYEGLRTNLPKEVMGYPDYSYPDDITESFITSNQVLEFLRSYAEHFKLKAHIKLQHEVIRVRPRL----DDWEVYVWD

                                      110       120       130       140       150       160       170       180       190       200
                              ....|....|....|....|....|....|....|....|....|....|....|....|....|....|....|....|....|....|....|
BmFMO2LR                      VITGEVFEENYDYVIVGNGHFSTPNMPNIRGEKLFKGTIIHSHDYRVPDVYKDRRVLVVGAGPSGMDIGLDVAECSKSLLHSHHS----KVNFRTPFPPH
HaFMO2LR                      VVTKETFEEDFDYIIVGNGHFSKPSYPNIPSEDLFTGRIIHSHDYKAPEPFTNRRVLVVGAGPSGMDIGLEVADVASALIHSHHS----KINWTTPFPPH
BmFMO1LR                      TDTKENVTETCDFIVVANGPYNTPVWPKYDGIDTFEGSMIHSHDYKDRKAYKNRKVLIVGAGASGLDLAIQLSNVTAKLVHSHHL-----VYNEPKFFDG
HaFMO1LR                      TDTKEQLTEVCDFIVVASGEFSSPVIPNIDRLEMFKGKVIHSHDYKDAEEFRNRRVLLVGAGASGLDLAMQLSNVTSQLFHSHHL-----NYNQPDFSKT
Bicyclus-FMO1-PepPart         TDERGDHTVACDFVVVANGQVR-PVVPMYPGLDKYQGSIYHSHDYKAPEPYSDRRVLLVGAGASGLDLAVQLHNITEKLVHSHHL-----KYNQPNFSKR
AcFMO1pGEMproof+S             TGTKQNVSEECGFVVVANGEYTAPHVPYFAKQEDFKGKMLHSHDYRDSEDYRGLRVLVVGAGPSAFDLAAHLINVTSMFIHSHHL-----DAKIQKVYGN
AvFMO1hypLR                   TDTKKNVSEECGFVVVANGEYIVPHVPYFAKQEDFKGKILHSHDYRDSEDYRCLRVLVVGAGPSAFGLAAHLINVTSMFIHSHHL-----DANIQKAYGN
dsannioFMOint_3raceLR         TDTKHNVSEECGFVVVATGEYIAPHVPYFAKQEDFKGKMLHSRDYRDSEDYRGLRVLVVGAGPSAFDLATHLINVTSMFIHSHHL-----DASIEKVYGN
TjFMO2pGEMproof+S             TDTKENVSEECGFVVVANGEYIAPHIPYFAKQEDFQGKMPHSHDYRDSEDYRGLRVLVVGAGPSAFDLATHLINVTSMFIHSHHL-----DAKLPEVYGN
eacreaFMOint_3race_tempLR     TDTKQNVTEECGFVVVANGEFTAPHVPYFAKQEDFKGKMLHSHDYRDLEDYRGLRVLVIGAGPSAFDLAAHLVNVTSMFIHSHHL-----IADIQEVYGN
AcSNOoSproofLR                TDTRQNYTEECGFVVVATGEYSTPKIPHIKGQELYKGKTMHSHDYKDPEDFRGQRVMLIGAGPSGLDLAVQLSNVTSKLVHSQHIIKSFKIYNQPDFPGN
dsannioSNOint_3raceLR         TDTRQNYTEECDFIVVASGEYSTPKIPNIKGQELYKGKTMHSHDYKDPEDYRGQRVTLIGAGPSGLDLAVQLSKVTSKLVHSHHIIKSFKIYNQPDFPGN
GgSNOoSproofLR                TDTRKNYTEECDFVVVATGEYSTPKIPHIEGQELYTGKTMHSHDYKDPEDFRGQRVMLIGAGPSGLDLAVQLSNVTSKLVHSHHIIKSFKIYNQPDFPGN
eacreaSNOint+3raceLR          TDTRHNYTEQCDFVVVATGEYSTPKIPPIKGHEIYEGKTMHSHDYKDPEDFRGQRVMLIGAGPSGLDLAVQLSNVTSKLVHSHHIIKSFKIYNQPDFPGN
NoxTyriaproofLR-S             TDTRKNYTEECDFVVVASGEFSTPKIPHIKGQEEYKGKTMHSHDYKEAESFRGQRVLVIGAGPSGLDVVMQLSNITSKLVHSQHILKSWHIFNQPDFPGN
BmFMO3LR                      LLNGESETREYDYVFVCNGHYNTPFIPNIPGLKEFQGDVMHSHDYRVPEIFSGKRVLVVGAGPSGMDIALEVTNVAHKVILSHHL----KEQPRTVFPDN
HaFMO3bLR                     LLNGHSETREYDYVFVCNGHYNTPFIPQIPGLKEFQGDVMHSHDYRVPDIFTDKRVLVIGAGPSGMDIALELTSVSKKVILSHHL----KDQPRTVFPEN
Plodia-FMO3-PepPart           LRTGVSQTKEYDYIFVCNGHYNTPFIPSIPGLKEYEGDVMHSHDYRVPEIFSNKRVLVIGAGPSGMDIALEITNVTDKVILSHHL----KEQPKTVFPDN
FMO3006                       LVTNKIEFQYFDKVLVANGHYHTPNYSQIPNMERFKGQFLHSHDFRSREVFEGKSVLVIGAGPSGMDLSNIISRTADRVTISHHL----TDIGQHSFFEN
FMO3174                       HSTDTCDPVYYDFVYVCNGHYTEPDLPEVEGLDLFEGNKMHSHLYRKADKFKDARVLIIGAGPSGMDITNHVRLAAKQVFLSHHL----STTPNTAFMGN
```

```
                                            210       220       230       240       250       260       270       280       290       300
                                   ....|....|....|....|....|....|....|....|....|....|....|....|....|....|....|....|....|....|....|....|
BmFMO2LR                           YVRKPDVKEFNETGVIFVDGTYEEIDDVIYCTGFQYDYPFLD-KTCGLDIDPHSVVPLYKYMVNIRQPSMVILGLVVRACLVVALDAQARYATALIKGNFTL
HaFMO2LR                           YHKKPDIKEFNETGVIFEDGSFEEIDDVIYCTGFYYDFPFLD-ESSGLTMEPKSVVPLYRYTVNINQPSMFIMGAFIRACLVVALDAQARYATAYIKGNFSL
BmFMO1LR                           YVKKPDIMAFTPKGVIFRDESFEELDDVIFCTGYDFNHPFLD-ESCGVTSTAKFVLPLHKQLVNIKHPSMVFLGIAKKIIT-RVMDAQAEYAALLASGKLKL
HaFMO1LR                           YVKKPDIDSFTPTGAFFVDGSTEEFDDVIFCTGYNYNHPFLD-SSSGVTASRKFVLPLYQQTVNIKHPSMTFVGVSKKVIN-RVMDAQGQYAAALASGKFQL
Bicyclus-FMO1-PepPart              YVKKPDIHGFVENGVIFVDGSFEEVDDIIFCTGYECYHPFLD-ETSGLTRSGKYVMPLYQHIVNMRRPTMTFIGVVNKVIT-KVMDAQAAYIASLIAGKFKL
AcFMO1pGEMproof+S                  YKRKPDIKHFTPTGAVFVDDTTEEFDVAILCTGYRYSFPFLNYQSSGVTSSAKYIMPLYNQLININHPTMTFVGTGKYSIG-LVRDRQGHYSAQLAAGLVKL
AvFMO1hypLR                        YKRKPDIKHFTPTGAVFVDDSTEEFDVAILCTGYKYSFPFLNYESSGVTSSEKYIMPLYNQLININHPTMTFVGTGKYSIG-LVRDRQGHYSAQLAAGLVKL
dsannioFMOint_3raceLR              YKKKPDIKHFTPTGAVFVDDSTEEFDVAILCTGYAYTFPFLNYQSSGVTWAPKYIMPLYNQLININHPTMTFVGTGKYSIG-VVRDRQGHYSAQLAAGLVKL
TjFMO2pGEMproof+S                  YKRKPDIKHFTPTGAVFVDGSTEEFDVAILCTGYKYSFPFLNYKSSGVAWTDKYVMPLYNQLININYPTMTFVGTGKYSIG-LVRDRQGHYSAQLAAGLVKL
eacreaFMOint_3race_tempLR          YKHKPDIKHFTPTGAVFVDNSTEEFDVAILCTGYTYSFPFLNYQSSGVTWSKEYIMPLHNHLININHPTMTFVGAAKYTLG-LVRDRQGHYSAQLAAGLIKL
AcSNOoSproofLR                     YISKPNVKYFTPNGAVFEDDTSEEFDIIIYCTGFYYNHPFLSTQSSGVTTTENYVMPLYQAVVNINQPTMTFIGICKPGFA-KILDLQAQYSAALAAGQFKL
dsannioSNOint_3raceLR              YISKPNVKYFTPNGAVFEDGTSEDFDLVIYCTGFYYNHPFLSTQSSGVTLTENYVMPLYQQVVNINQPTMTFIGICKPFFA-KLLDLQAQYSAALAAGRFKL
GgSNOoSproofLR                     YISKPNVKYFTSTGAVFEDGTTEDFDIVIYCTGFYYNHPFLSTQSSGVTTTENYVMPLYQAVVNINQPTMTFVGICKPFYA-KILDIQAQYSAALAAKKFEL
eacreaSNOint+3raceLR               YISKPNVKHFTSNGAVFEDGTTEEFDLVIYCTGFYYSHPFLSTQSSGIIATENYVMPLYQEIVNINQPTMTFIGICKPFFA-KILDIQAQYSAALAAAQFKL
NoxTyriaproofLR-S                  FISKPNVKHFTANGAVFEDDTVEEFDMVIYCTGFYYNHPFLSTLSSGITATENYVMPLYQQVVNINQPTMTFVGICKPFFA-KLLDQQAHYSAKLAAGHFKL
BmFMO3LR                           LTQKPDVKRLDGKKVHFADESEDEVDVVFLCTGYLYNFPFLH-ESCNISVEDNCVEPLYKHLVNIHHPTMCFIGVPYYVCAFSMFDLQVRYYIRSINGTFSL
HaFMO3bLR                          LEQKPDVERLDGHKACFLDGTEDEVDVVFLCTGYLYNFPFLH-ESCGIVVEDNCVEPLYKHVVNMNHPSMCFIGVPYYVCAFSMFDLQVRYYVRSMNGTFSL
Plodia-FMO3-PepPart                LVQKPDVXXXXXXXXXXXXXXXXXXXXXXXXXXXXXXXXXXXXXXXXXXXXXXXXXXXXXXXXXXXXXXXXXXXXXXXXXXXXXXXXXXXXXXXXXXXXXXXX
FMO3006                            VQQKPDVRELDEKGAFFVDGSYQEFDTVFFCTGYKYAFPFLT-VDSGIYVEDNYVQELYKQCINIRNPSMALIGLPFYVCAAQMMDIQARFIMSYYNGSNEL
FMO3174                            VTQKPDVKRFTKDGAVFTDGSTESFDHVMFCTGYKYTFPCLS-TDVGVQVIDNFVQPLWKHCININHPTMAFVGLPFNVIPTHIFDMQVRFTLKFFTGQRKF
```
