# Supplementary material for: Flavin-Dependent Monooxygenases as a Detoxification Mechanism in Insects: New Insights from the Arctiids (Lepidoptera)
Source: PLoS One. 2010 May 3;5(5):e10435. doi: 10.1371/journal.pone.0010435 (PMC2862711; doi:10.1371/journal.pone.0010435)
Supplement: Table S1 — Sequences of primers used for the identification and cloning of cDNAs of flavin-dependent monooxygenases of the Lepidoptera. (0.02 MB PDF) [file pone.0010435.s002.pdf]

No.	Primer Sequence	Properties
P01	5′-dGTC GAC TCG AGA ATT C(T)$_{17}$-3′	oligo(dT) primer
P02	5′-dTG**Y** **R**T**N** AT**H** GG**N** GC**N** GG-3′	degenerate primer
P03	5′-dCC**N** A**YR** AA**I** G**Y**C AT**N** GT**N** GG-3′	degenerate primer
P04	5′-dGTG ACA ATT GGA AGG TGA CAT ATA T-3′	*Ac*FMO 3’RACE
P05	5′-dCTG AAT TTT AGC ATC TAA A-3′	*Ac*FMO 5’RACE
P06	5′-dTTG ATG AGG TGA GCA GCA AGG TCA AA-3′	*Ac*FMO 5’RACE
P07	5′-dTCG TGA CTG TGT AGC ATC TTT CCT TTA-3′	*Ac*FMO 5’RACE
P08	5′-dCC**N** AC**N** ATG **R**C**I** TT**Y** **R**T**N** GG-3′	degenerate primer
P09	5′-dGTA AGG TAC ATG GGG CAC AAT-3′	*Av*FMO 5’RACE
P10	5′-dGAC TTC GGA ATT GAA TAT ATT TTT CCA AAT-3′	*Av*FMO 5’RACE
P11	5′-dGGC AAT TCC ATC GTC GGA TAG AAA GA-3′	*Av*FMO 5’RACE
P12	5′-dGG**N** GG**N** AC**I** TGG **M**G**N** TA**Y**-3′	degenerate primer
P13	5′-dAGA CTT TCC TGG CAA CTA CAT AAG TAA-3′	*Gg*PNO 3’RACE
P14	5′-dCAA TTT CGA GGT GAC GTT AGA-3′	*Gg*PNO 5’RACE
P15	5′-dTAA TTC TTG TCC TTC GAT GTG AGG TAT TT-3′	*Gg*PNO 5’RACE
P16	5′-dTGA ATG TTG TCC ATC AAT TTA AAA TGT CTT A-3′	*Gg*PNO 5’RACE
P17	5′-dGG**R** AA**R** TC**N** GG**Y** TG**R** TT**R** **W**A-3′	degenerate primer
P18	5′-dCTC ACA TCA AAG GAC AAG AGT TAT A-3′	*Ac*PNO 3’RACE
P19	5′-dCAC TAA TTT CGA GGT GAC GTT AGA AA-3′	*Ac*PNO 5’RACE
P20	5′-dTGT ATC CCG TCC ATT CAA CCC AAT T-3′	*Ac*PNO 5’RACE
P21	5′-dGCT GAT AAA CGA ACG AGT ACC ATT T-3′	*Ac*PNO 5’RACE
P22	5′-dACA AAA TCA CAT TCT TCC GTA TAA-3′	*Av*PNO 5’RACE
P23	5′-dGGT TCC AAC TGT CTC CCG TCC ATT CT-3′	*Av*PNO 5’RACE
P24	5′-dGAA AGA TTT AAT ATA CTT ATA GAA GCA ATC-3′	*Av*PNO 5’RACE
P25	5′-dGGT GGG TGA CAA TTG GAA CGT AAC ATA TA-3′	*Ds*FMO 3’RACE
P26	5′-dGGG AGA CAG TTG GAA CCT AAC GTA TAT-3′	*Ds*PNO 3’RACE
P27	5′-dCGT TGT TCG TGG CCA ACG GGG AAT TT-3′	*Ea*FMO 3’RACE
P28	5′-dCGT TCG TCA CTG GAG GTT GCT TCT ATA A-3′	*Ea*PNO 3’RACE
P29	5′-dTG**R** TG**N** **SWR** TG**D** AT**R** AAC AT-3′	degenerate primer
P30	5′-dGTA TAA GAA CTT GAG AAC AAA CTC GTT T-3′	*Tj*FMO 3’RACE
P31	5′-dATG GGC CGG CCC CAA CTA-3′	*Tj*FMO 5’RACE
P32	5′-dCGA AGT AAG GTA TAT GTG GTG CGA TGT A-3′	*Tj*FMO 5’RACE
P33	5′-dTCT CCC ACC CGC TCA ACA CCA GTT A-3′	*Tj*FMO 5’RACE
P34	5′-dTAT ACA TAT GCA ATC TGA CCA TGC GTC TCG AGC AT-3′	*Gg*PNO expr. constr.
P35	5′-dTAT AGG ATC CCT AAT GAT GAT GAT GAT GAT GAT CGT CAA AAG GAC ATA TTT CCT TCC TT-3′	*Gg*PNO expr. constr.
P36	5′-dTAT ATA GGT CTC CTC GAG AAA AGA AGT TCA GCG TCT CGA GTA TGC ATT ATT-3′	*Tj*SNO RT-PCR
P37	5′-dTAT AGA TCT CTA ATG GTG ATG GTG ATG GTG ATC GTC ATA GGG ACA TAC TTC TTC-3′	*Tj*SNO RT-PCR
P38	5′-dTAT ACA TAT GGG TGA TAC GTT CCC GCA AGA AAC AA -3′	*Tj*FMO RT-PCR
P39	5′-dTAT ACT CGA GTA CTT GTA CAT CTA AAG GAC ATA CTT TTT TCT-3′	*Tj*FMO RT-PCR
