# Supplementary material for: Flavin-Dependent Monooxygenases as a Detoxification Mechanism in Insects: New Insights from the Arctiids (Lepidoptera)
Source: PLoS One. 2010 May 3;5(5):e10435. doi: 10.1371/journal.pone.0010435 (PMC2862711; doi:10.1371/journal.pone.0010435)
Supplement: Table S2 — PCR-based strategy for identification of partial FMO-like sequences of various lepidopteren species. (0.01 MB PDF) [file pone.0010435.s003.pdf]

*Av*PNO	*Arctia villica*<br>putative PNO	internal fragment of ca. 600 bp<br>- nested PCR<br>→ 1. PCR: annealing: touch-down 60 → 45°C<br>primer pair P12/P1<br>→ 2. PCR: annealing: touch-down 60 → 45°C<br>primer pair P12/P17<br>5'-RACE: primers P22-P24
*Ds*FMO	*Diacrisia sannio*<br>putative FMO	internal fragment of ca. 800 bp<br>- PCR: annealing: touch-down 60 → 45°C<br>primer pair P12/P3<br>3'-RACE primer pair P25/P1
*Ds*PNO	*Diacrisia sannio*<br>putative PNO	internal fragment of ca. 480 bp<br>- PCR: annealing: 57°C<br>primer pair P12/P17<br>3'-RACE primer pair P26/P1
*Ea*FMO	*Estigmene acrea*<br>putative FMO	internal fragment of ca. 780 bp<br>- PCR: annealing: touch-down 65 → 50°C<br>primer pair P12/P3<br>3'-RACE primer pair P27/P1
*Ea*PNO	*Estigmene acrea*<br>putative PNO	internal fragment of ca. 580 bp<br>- PCR: annealing: touch-down 65 → 50°C<br>primer pair P12/P17<br>3'-RACE primer pair P28/P1
