# Supplementary material for: Flavin-Dependent Monooxygenases as a Detoxification Mechanism in Insects: New Insights from the Arctiids (Lepidoptera)
Source: PLoS One. 2010 May 3;5(5):e10435. doi: 10.1371/journal.pone.0010435 (PMC2862711; doi:10.1371/journal.pone.0010435)
Supplement: Table S3 — Accession numbers of all nucleotide sequences that have been identified within this project and that have been taken from the databases. (0.01 MB PDF) [file pone.0010435.s004.pdf]

Sequence name		Accession number
AcFMO	*Arctia caja*	FN649426
AcPNO	*Arctia caja*	FN649422
AvFMO	*Arctia villica*	FN649427
BaFMO1	*Bicyclus anynana*	GE660293
BmFMO1	*Bombyx mori*	GU564654
BmFMO2	*Bombyx mori*	GU564656
BmFMO3	*Bombyx mori*	GU564657
DmFMO3006	*Drosophila melanogaster*	NP_611859
DmFMO3174	*Drosophila melanogaster*	NP_610217
DsFMO	*Diacrisia sannio*	FN649428
DsPNO	*Diacrisia sannio*	FN649423
EaFMO	*Estigmene acrea*	FN649430
EaPNO	*Estigmene acrea*	FN649425
GgSNO	*Grammia geneura*	FN649424
HaFMO1	*Helicoverpa armigera*	GU564659
HaFMO2	*Helicoverpa armigera*	GU564660
HaFMO3	*Helicoverpa armigera*	GU564662
PiFMO3	*Plodia interpunctella*	EB827095, EB823265
TjFMO	*Tyria jacobaeae*	FN649429
TjSNO	*Tyria jacobaeae*	AJ420233
